# Correlates of domestic violence perpetration reporting among recently-married men residing in slums in Pune, India

**DOI:** 10.1371/journal.pone.0197303

**Published:** 2018-05-17

**Authors:** Ameeta S. Kalokhe, Sandhya R. Iyer, Keshav Gadhe, Tuman Katendra, Anuradha Paranjape, Carlos del Rio, Rob Stephenson, Seema Sahay

**Affiliations:** 1 Emory University School of Medicine, Department of Medicine, Division of Infectious Diseases, Atlanta, Georgia, United States of America; 2 Emory University Rollins School of Public Health, Department of Global Health, Atlanta, Georgia, United States of America; 3 National AIDS Research Institute, Department of Social and Behavioral Research, Pune, India; 4 Temple University Lewis Katz School of Medicine, Department of Medicine, General Internal Medicine, Philadelphia, Pennsylvania, United States of America; 5 University of Michigan, School of Nursing, Department of Health Behavior and Biological Sciences, Ann Arbor, Michigan, United States of America; University of Westminster, UNITED KINGDOM

## Abstract

Domestic violence (DV) is prevalent in low-income and slum-dwelling communities in India. To date, the focus of DV prevention in resource-poor settings has largely been with women. We herein aim to identify correlates of DV perpetration to help inform future primary prevention efforts that focus on behavioral change in men. Utilizing a cross-sectional design, potential correlates of DV perpetration were explored among a geographically-clustered random sample of 100 recently-married men residing in slums in Pune, India. In multivariable regression, DV perpetration was associated with less time spent alone in the relationship post-marriage (standardized β = -0.230, p<0.01), not attaining the “husband ideal” (standardized β = -0.201, p<0.05), poor resilience (standardized β = -0.304, p < .01), having limited definitions of behaviors constituting DV (standardized β = -0.217, p<0.05), and reporting greater jealousy if the participant’s spouse were to talk to men outside the family (standardized β = 0.272, p<0.01). The identified correlates should inform components of future DV primary prevention interventions that target men as potential perpetrators or the couple as a unit.

## Introduction

In India, domestic violence (DV) is defined as the physical, sexual, and psychological abuse and control against a woman by a partner *or* family member [1]. While DV is prevalent globally [2,3], approximately one out of every three women in India report experiencing violence at the hands of their spouse at some point in their lifetime [4]. Several studies demonstrate that this proportion is even greater in slum-dwelling and other low-income communities across India [5–13]. Proposed explanations for higher DV reporting among slum-dwelling communities include heightened stress and conflict due to poverty, overcrowding, and associated conditions, weakened support systems, stronger norms accepting DV, and poverty-related perceived shortcomings in achieving the masculine ideal leading men to feel the need to prove dominance over those more vulnerable, often their spouses [14,15].

Developing strategies to curb DV is critical not only because DV impinges on human rights, but also because it negatively affects the mental and physical health of the survivor and her family. Women who experience DV report higher rates of mental health disorders including depression, anxiety, post-traumatic stress disorder and suicidal ideations [16,17]. Further, they incur higher risk of sexually transmitted infections including HIV, pain disorders, and cardiovascular, respiratory, reproductive, and gastrointestinal disease [17–20]. And, their children are more likely to have behavioral and learning difficulties, emotional problems, die at a young age, and themselves experience or become perpetrators of DV [21–23].

To date, in resource-limited settings, the focus of primary DV prevention has been with women, although recent interventions have begun to engage boys and men to prevent DV [24–29]. Unfortunately, little is known about determinants of DV perpetration by men in low- and middle-income countries (LMICs), particularly in South-East Asia where DV prevalence is known to be exceptionally high [3], and among those residing in slum communities where DV is reported most commonly. While it would seem natural that the determinants of DV perpetration would parallel those of DV experience, where the bulk of LMIC literature exists, such studies tend to solely explore the woman’s perspective of DV risk.

The bulk of literature examining correlates of DV perpetration comes from high-income settings and has linked DV perpetration to the following: young age, low socio-economic status, alcohol and substance abuse, stress, having a mental health or personality disorder, poor social support, experiencing abuse as a child, witnessing or experiencing DV oneself, accepting attitudes toward DV, frontal lobe dysfunction and hormonal and neurotransmitter imbalance, marital discord, relationship dissatisfaction, and jealousy[30–32]. The few studies examining perpetration of DV in India and other LMIC settings suggest DV perpetration is associated with age, low socio-economic status, caste, religion, urban residence, accepting attitudes toward wife beating, childhood witness of DV, aggression in the workplace or community, alcohol use, having multiple children, larger family dwelling (i.e. joint families), marital duration, marital conflict (over sex and the male partner’s infidelity), and failure of the wife to bring sufficient dowry[33–37]. Strong patriarchal norms and the caste system also operate in violence perpetration by men. There remains a large gap in exploring causes of DV perpetration in low-income populations in LMIC settings where effects of poverty, stress, and powerlessness are amplified.

As part of the formative work in developing a couples-based intervention for the primary prevention of DV in India, we explored potential determinants of DV perpetration among recently-married men residing in slum communities. This is an important population in whom to study determinants of DV because there is often minimal acquaintance pre-marriage, social dynamics and employment constraints heavily limit the time they spend together post-marriage, crowding, poverty, and powerlessness likely further fuel DV perpetration, and involvement by family members in the marriage is substantial (regardless of residence in joint versus nuclear families).

## Methods

### Ethics statement

This study was approved by the Institutional Review Board of Emory University (Atlanta, Georgia, USA, IRB00069846) and the Ethics Committee of the National AIDS Research Institute (NARI, Pune, India, NARI-EC/2013-28). All participants provided written informed consent prior to taking part in the study. The study protocol was developed in consultation with the WHO guidelines for the ethical conduct of DV research.[38]

### Study setting

The study was undertaken in Pune, the ninth most populous city in India with a population of 3.1 million, located in the western state of Maharashtra. Per the 2011 Census of India,[39,40] the female: male: sex ratio is 0.948 and the literacy rate is high (92% in men and 87% in women). Approximately one-fourth (22%) live in slums. While city-specific data is not available, Government of Maharashtra estimates suggest that 32% of the state’s urban population lives below the poverty line, and the mean age of marriage for girls is 20.6 years. The only published study evaluating DV prevalence in Pune estimated lifetime physical DV in slum-dwelling women to be 62%.[12]

Study interviews were conducted in private in one-on-one face-to-face interviews. Participants were requested to come to a nearby site designated by the research staff (i.e. a partner NGO site or NARI clinic) for the interview. However, many were unable and/or unwilling to leave their homes and/or communities for the interview. For such participants, the interview was conducted in a location of their choice provided that they could assure with confidence that privacy would be maintained for the 2 hours necessary to complete the study visit. Such participant-selected venues included their homes, shops, the back seats of parked *rickshaws* (three-wheeled, hooded vehicles), nearby parks, cow pens, and community libraries.

### Study design

The study utilized a cross-sectional design wherein a semi-structured questionnaire was administered by study staff members to the participants in one-on-one, face-to-face interviews. Interested potential participants meeting the study eligibility criteria (stated below) provided written informed consent and then completed a 182-item, interviewer-administered questionnaire. The questionnaire included items about past 3-month perpetration of DV as well as various potential socio-behavioral determinants of DV perpetration. Interviews were conducted in the participant’s language of choice (i.e. Hindi, Marathi, or English), completed in one study visit, and of 60–120 minutes duration. Upon completion of the interview a debriefing session was held during which the participant could elaborate on his responses and further ask questions.

### Participant recruitment and enrollment

Eligibility criteria included being a man 1) age 18 or older, 2) married for 3–15 months, 3) in a first marriage, 4) residing with his spouse, 5) living in a slum in either the Pune Municipal Corporation (PMC) or Pimpri-Chinchwad Municipal Corporation (PCMC) area of Pune City and planning to reside in the area for the majority of the following 12 months, and 6) oral fluency in either Marathi, Hindi, or English.

As this male perpetration study is part of a larger study that also independently explored determinants of DV experience in recently-married women, measures were taken during recruitment to ensure men and women were never recruited from the same slum area within a ward. Recruitment involved a 2-stage process: 1) geographically-clustered, random sampling whereby slum areas were sampled from the 21 PMC and PCMC geographic wards; 2) convenience sampling at the slum level wherein 4–5 men were recruited from each slum area. In situations where insufficient eligible and willing participants were found in one slum area, additional participants were recruited from a slum in the adjacent ward. Field staff employed various strategies to recruit potential participants: door-to-door recruitment and snowball sampling (during which families were asked whether they had a newly-married man in their household or knew of a newly-married man in their community who might be interested in participating), community meetings, and recruitment in coordination with *anganwadis* (government childcare centers), non-governmental organizations (NGOs) which serve slum communities, and slum-based *mitra mandals* (male social clubs).

### Data collection

The survey instrument (S1 Appendix) was developed in consultation with existing global literature about DV determinants, pre-tested in 5 volunteers, and modified based on pre-testing results prior to administration. The primary outcome of prior 3-month DV perpetration was measured using an adapted version of the 63-item Indian Family Violence and Control Scale (IFVCS).[41] The IFVCS is a 63-item, culturally-tailored measure that was developed to measure physical, sexual, psychological abuse and control *experience* among married women. Its development was informed by qualitative interviews with lay community members and individuals working directly with DV survivors in Pune and it has been validated in a sample of married women in Pune previously.[41] To measure perpetration, the IFVCS umbrella question was replaced with a question that queried how often the participant or his family had committed each of the 63 acts of violence against his wife in the prior 3-month period.

Potential predictors of DV perpetration were assessed across 6 major domains: 1) socio-demographics, 2) DV conceptualization and acceptance, 3) the marital relationship and marital family relationship, 4) sexual communication and behaviors, and sexual and reproductive health, 5) substance abuse and gambling, and 6) stress, resilience, and social support. The items assessed in each domain are included in Table 1.

**Table 1:** Correlates of DV perpetration among recently-married men living in Pune slum communities (n = 100).

Potential correlate	No. (%)	Correlation with DV total	Retained in domain model
*Domain 1*: *Socio-demographics*			
Age, mean (SD), years^†^	25.75 (2.38)	-0.126	
Age of spouse, mean (SD), years^†^	20.98 (2.40)	-0.151	
Age gap (spouse-self), mean (SD), years^†^	-4.77 (2.40)	-0.027	
Education		-0.080	
≤ Primary (7^th^ standard)	12 (12)		
Secondary (8^th^-10^th^ standard)	43 (43)		
≥ Higher secondary (≥11^th^ standard)	45 (45)		
Additional training	18 (18)	0.078	
Education of spouse		-0.115	
≤ Primary (7^th^ standard)	14 (14)		
Secondary (8^th^-10t standard)	39 (39)		
≥ Higher secondary (≥11 standard)	47 (47)		
Additional training by spouse	12 (12)	-0.131	
Employment	93 (93)	-0.067	
Employment of spouse	8 (8)	-0.054	
Monthly income		-0.116	
None	8 (8)		
Rs. 0 <x≤ 8000	13 (13)		
Rs. 8000<x≤10,000	36 (36)		
> Rs. 10,000	43 (43)		
Monthly income of spouse		-0.016	
None	91 (91)		
Rs. 0 <x≤ 8000	6 (6)		
Rs. 8000 <x≤ 10,000	2 (2)		
> Rs. 10,000	1 (1)		
Family type pre-marriage: nuclear	4 (4)	-0.110	
Family type post-marriage: nuclear	9 (9)	0.008	
Household members, mean (SD)	5.72 (2.55)	0.252*	**X**
Caste, reserved	67 (67)	0.032	
Religious affiliation			
Hindu	72 (72)	-0.122	
Buddhist	16 (16)	0.182	
Muslim	10 (10)	-0.070	
Christian	2 (2)	0.028	
Spouse ever pregnant	54 (54)	0.012	
Had livebirth(s)	14 (14)	-0.059	
Had planned abortion(s)	3 (3)	-0.129	
Had unplanned abortion(s)	7 (7)	0.033	
Spouse currently pregnant	32 (32)	0.102	
*Domain 2*: *DV conceptualization and acceptance*			
Household decision-making: mainly wife^†^	0.14 (0.11)	0.077	
Household decision-making: both^†^	0.50 (0.21)	**-0.189**^**+**^	
Situational acceptance of wife-beating^†^	0.17 (0.16)	0.232*	
Situational acceptance of wife’s sexual refusal^†^	3.48 (0.71)	**-0.194**^**+**^	**X**
Liberal definition of items constituting DV^‡^	3.47 (0.40)	-0.445***	**X**
Acknowledgment of DV occurrence in a friend/relative^‡^	0.52 (0.30)	-0.220*	
*Domain 3*: *The marital relationship and marital family relationship*			
Marital duration, mean (SD), months^†^	9.03 (3.52)	-0.062	
Marriage type: arranged	68 (68)	-0.060	
Marriage within caste	88 (88)	-0.260	
Marriage within family	59 (59)	0.043	
Total face-to-face time with partner alone pre-marriage		-0.137	
None	24 (24)		
< 1 month	46 (46)		
1–6 months	8 (8)		
> 6 months	21 (21)		
Total time in contact with partner pre-marriage		-0.046	
None	10 (10)		
< 1 month	22 (22)		
1–6 months	32 (32)		
> 6 months	33 (33)		
Extent of acquaintance with partner pre-marriage		-0.228*	
Not at all	12 (12)		
Very little	13 (13)		
Somewhat	19 (19)		
Great extent	53 (53)		
Time spent with partner alone each week post-marriage		-0.202*	**X**
Never	5 (5)		
Weekends/holidays only	4 (4)		
At least 3–4 days/week	88 (88)		
Greatest time spent working towards dreams of			**X**
Spouse	8 (8)	-0.057	
Self	19 (19)	0.211*	
Both	66 (66)	-0.202*	
Don’t	2 (2)	0.076	
Greatest time spent discussing things of interest to			
Spouse	16 (16)	0.184	
Self	10 (10)	0.001	
Both	71 (71)	-0.199*	
Great time spent doing things of interest to			
Spouse	19 (19)	0.120	
Self	10 (10)	-0.021	
Both	68 (68)	-0.136	
Extent of attainment of the “husband ideal”		-0.243*	**X**
≤ Very little	8 (8)		
Somewhat	30 (30)		
Great extent	59 (59)		
Extent of spouse’s attainment of the “wife ideal”		-0.216*	**X**
≤ Very little	8 (8)		
Somewhat	16 (16)		
Great extent	73 (73)		
Satisfaction with future spouse at time of marriage		0.058	
≤ Somewhat	12 (12)		
Great extent	84 (84)		
Satisfaction with maanpaan (wedding-related gifts) at time of marriage		-0.107	
≤ Somewhat	7 (7)		
Great extent	84 (84)		
Familial satisfaction with maanpaan (wedding-related gifts) at time of marriage		**-0.196**^**+**^	
≤ Somewhat	15 (15)		
Great extent	77 (77)		
Satisfaction with in-law’s treatment since marriage		-0.130	
≤ Very little	7 (7)		
Somewhat	14 (14)		
Great extent	75 (75)		
Parent’s satisfaction with spouse as a daughter-in-law		-0.216*	
≤ Somewhat	10 (10)		
Great extent	86 (86)		
Conflict negotiation skills (CTS2n) ^‡^	3.28 (0.64)	-0.151	
Extent of jealousy if spouse talks to men within family		0.265**	
Never	83 (83)		
Rarely	5 (5)		
≥Sometimes	8 (8)		
Extent of jealousy if spouse talks to men outside family		0.331***	**X**
Never	68 (68)		
Rarely	10 (10)		
≥Sometimes	18 (18)		
*Domain 4*: *Sexual communication and behaviors*, *and sexual and reproductive health*			
Confidence in knowledge about sexual intercourse		-0.070	
≤Very Little	5 (5)		
Somewhat	32 (32)		
Great extent	57 (57)		
Capacity to communicate unwillingness to have sex with partner		-0.257*	**X**
Very little	4 (4)		
Somewhat	8 (8)		
Great extent	84 (84)		
Capacity to communicate willingness to have sex with partner		**-0.194**^**+**^	
Very little	3 (3)		
Somewhat	8 (8)		
Great extent	85 (85)		
Last sexual intercourse		0.092	
Persuaded partner or partner persuaded	16 (16)		
Mutually willing (baseline group)	79 (79)		
Forced my partner	1 (1)		
Prior use of a contraceptive	29 (29)	-0.050	
Prior discussion of contraceptive use with partner	46 (46)	-0.064	
Engagement in sexual relations outside of spouse	21 (21)	**0.170**^**+**^	
*Domain 5*: *Recent substance abuse and gambling*			
Prior 3-month alcohol use		0.115	
Never	59 (59)		
Rarely	17 (17)		
Sometimes	13 (13)		
Often	8 (8)		
Prior 3-month drug use	3 (3)	0.342***	**X**
Prior 3-month betting/gambling	5 (5)	0.091	
Domain 6: Stress, resilience, and social support			
Stress due to financial trouble	63 (63)	-0.035	
Stress due to non-continuous employment	36 (36)	0.112	
Average number of scenarios causing stress	0.22 (0.14)	-0.041	
Perceived stress in past 3 months		0.016	
Never	8 (8)		
Rarely	22 (22)		
Sometimes	52 (52)		
Often	15 (15)		
Resilience^‡^^√^	3.72 (0.59)	-0.405***	**X**
Greatest support person if stressed: spouse	33 (33)	-0.018	
Support spouse if she’s in conflict with family		0.006	
≤ Rarely	22 (22)		
Sometimes	37 (37)		
Often	33 (33)		
Greatest support person(s) if marital conflict			
Parents	80 (80)	-0.134	
Parents-in-law	2 (2)	-0.177	
Other	13 (13)	0.037	
Perceived support from family if marital conflict		-0.138	
≤ Very little	6 (6)		
Somewhat	6 (6)		
Great extent	84 (84)		

Column 1 describes the potential correlates that were assessed, Column 2 the distributions of the correlates, Column 3 the correlation for the respective bivariate analysis, and Column 4 indicates whether the correlate was ultimately retained in the respective domain model (which was run using variables significant at the bivariate level, choosing between highly collinear variables within the domain). Significant correlations are noted as follows

^+^p<0.10

*p≤0.05

**p≤0.01

***p≤0.001.

Where test statistics are not followed by p-values, the correlations were not deemed significant. Variables designated with a † were analyzed as continuous variables, those designated with a “‡” were analyzed as available case means, and the remaining variables were categorical.

^√^In measuring “resilience,” we used the Connor-Davidson operational definition, “the personal qualities that enable one to thrive in the face of adversity,” and the Connor-Davidson Resilience Scale 10.

Most questions were drawn directly or adapted from validated scales. To assess how the participants conceptualized DV, we requested they specify the extent to which they considered each of the 63 IFVCS items [41] as violence (not violence, mild violence, moderate violence, or severe violence). To measure their awareness of DV occurrence in their close acquaintances, we asked whether they were aware of the 63 IFVCS items happening to a married woman among their relatives or friends. To assess attitudes toward gender-based household decision-making, we used the 4-item NFHS-3 decision-making module [42] plus 4 additional items surveying who should have the greater say in the healthcare of the wife, whether to have children, and whether and which contraception to use. The 7-item NFHS-3 Attitudes Toward Wife Beating questions [5] plus 3 additional items regarding whether physical violence was justified if the woman broke something expensive, was unable to have a child, or unable to have male child were used to assess attitudes toward DV acceptance. To assess attitudes toward a woman’s autonomy in sexual intercourse, the 3-item NFHS-3 “Attitudes Toward Refusing Sexual Intercourse with Husband” questions [5] were used. Lastly, the 6-item Conflict Tactics Scale-2 Negotiation Subscale[43] was used to measure husband-wife conflict resolution skills and the Connor-Davidson Resilience Scale 10 to measure resilience.[44]

### Participant and study team safety

The study field protocol was developed in consultation with the WHO guidelines about the safe and ethical conduct of DV research. Only one member per household was enrolled. Upon approaching a household, the study was first introduced as a ‘healthy relationship study,’ but at the time of informed consent provision, study staff informed the potential participant about the entirety of the interview components including DV questions. During the consent process participants were informed that if someone were to enter during the course of the interview, the interviewer would either stop the interview or switch topics to an unrelated health topic.

Prior to study initiation, all field staff, who were masters-level trained personnel, underwent a week-long training about interview conduct, confidentiality and research ethics, and participant and personal safety. To also optimize their field safety, study staff notified the nearest police station of the planned recruitment prior to entering a slum community, utilized community key informants (i.e. *anganwadi* workers, staff from NGOs with whom NARI has partnered for assistance with recruitment and retention in prior research) when available to enter the community, and electronically messaged the entire study team when they entered and exited an interview and the field. Additionally, female interviewers conducted recruitment in pairs. Lastly, weekly debriefing meetings with staff were held to discuss their field experiences and assess the resultant impact of on their personal emotional wellbeing.

### Data management and statistical analysis

Data was entered into Microsoft Access, cleaned in Microsoft Excel, and then transferred to SPSS for analysis. Table 1 footnotes detail how each variable was operationalized. Measures of DV perpetration, definition, and experience by family and friends as well as resilience and conflict negotiation skills were calculated as available case means across all items of the respective scales to avoid most missing data scenarios. For the primary outcome variable of DV perpetration, as well as knowledge of other’s DV experience, this represents a proportion because all items were binary. For DV definition, this represents an average that can be referred back to the ordinal scale for comparisons of severity (mild, moderate, etc). Related items among the predictors (such as the stress scale etc.) were combined in a similar fashion. Distributions of each of the potential DV predictors and outcome variable (past 3-month DV perpetration) were first assessed to justify the use of linear regression for DV perpetration and identify potential outliers (overly influential points) among predictors. Next significant correlates of DV perpetration were identified through bivariate analysis. All significant variables at the bivariate stage (p<0.05) were then examined within each domain for collinearity by examining the correlation matrix and looking for redundancy among predictors. If any two (or more) variables were considered collinear variable selection was based on the higher of the two correlations with the outcome. The next stage of data reduction was to allow the remaining significant predictors of DV perpetration to compete, within each domain, using multivariable regression models. The purpose of this was to find the most significant and *independent* predictors of DV perpetration within each domain before the domains were combined to complete a final model. This assures that only the most robust predictors are considered. Lastly, all variables significant in the domain-level models were included in a composite multivariable model with the exception of drug use as it was reported with extremely low frequency. Backward elimination was then used to eliminate non-significant variables, resulting in the final DV perpetration prediction model. Control variables “age” and “education” were also included for comparability with other studies.

## Results

### Study participants

A random, geographically-clustered sample of 100 recently-married men were enrolled from slums in Pune (Table 1). The average age was 25.75 years (σ = 2.38), greater than half (55/100) had not completed education beyond the 10^th^ standard, the vast majority were employed (93/100), but 57/100 had monthly income ≤ Indian Rupees (INR) 10,000. Most lived in joint families pre- and post-marriage (95/100 and 91/100, respectively), with an average of 5.72 (σ = 2.55) household members. The sample was largely of Hindu religion (72/100) and of reserved caste (67% or 67/100).

In contrast, the average age of the participants’ spouse was 20.98 years (σ = 2.40), and while the spouse’s education level was similar to that of the men (53/100 not completing education beyond 10^th^ standard), only 8/100 were employed, with the vast majority 91/100 having no income. Since marriage (i.e. ≤18 months) over half (54/100) had been pregnant and one-third (32/100) of spouses were reportedly pregnant at the time of their husband’s interview.

The average IFVCS scores (proportion of total DV types surveyed) for past 3-month perpetration of control, psychological, physical, and sexual DV were 0.63 (σ = 0.50), 0.06 (σ = 0.09), 0.01 (σ = .03), and 0.01 (σ = 0.03), respectively.

### Correlates of DV perpetration

In bivariate analysis (Table 1, Column 3), among all Domain 1 demographic factors DV perpetration was only significantly associated with having greater number of household members (p≤0.05). In the DV conceptualization and acceptance domain (Domain 2), DV perpetration was associated with less mutual household decision-making (p<0.10), less acceptance for a woman to refuse sex with her husband (p<0.10), limited acknowledgment of what constitutes DV (p ≤0.001), lack of knowledge of DV occurring in a friend or relative (p≤0.05), and reporting more situations in which it was justifiable for a husband to beat his wife (p≤0.05). Among the family relationship variables (Domain 3), DV perpetration was associated with the participant reporting he knew his partner less well at the time of marriage (p≤0.05), less time spent with his spouse alone post-marriage (p≤0.05), less satisfaction with the *maanpaan* received at marriage (p<0.10), less satisfaction of the parents with his wife as a daughter-in-law (p≤0.05), and not having attained what society expected of a husband (p≤0.05) and wife (p≤0.05). DV perpetration was associated with prioritizing time to work toward his own dreams and goals, and similarly not prioritizing time on mutual goals (p≤0.05), not prioritizing time to discuss mutual interests (p≤0.05), and reporting more jealousy if his wife talked to men either within or outside the family (p≤0.01, and p≤0.001, respectively). In the sexual communication and sexual health domain (Domain 4), DV perpetration was associated with less capacity to communicate to one’s spouse unwillingness and willingness to have sex (p≤0.05, and p<0.10, respectively), and having sexual relations with someone other than his spouse (p<0.10). Among the remaining variables, DV perpetration was associated with prior 3-month substance abuse (p≤0.001) and less resilience (p≤0.001).

### Building the DV perpetration model

Only a small number of variables were dropped from consideration due to collinearity: specifically not prioritizing time to work on dreams and goals was associated with working toward his own dreams and goals, so not prioritizing time was used for further model selection. Similarly jealously if the wife spoke to men within and outside the family were highly correlated, so only talking to men within was used as it was the higher correlated of the two.

Domain level models were next run using backward selection (p < 0.05) to determine the significant and *independent* predictors in each domain. Multivariable models for Domains 1 and 5 were not run because only one variable was a candidate. Prior 3-month drug use was excluded from the final model because of the low frequency of affirmative response (3% or 3/100). The final model is shown in Table 2. In the final model, DV perpetration was associated with limited acknowledgment of behaviors constituting DV, less time spent alone in the relationship post-marriage, not attaining the “husband ideal,” lack of resilience, and reporting greater jealousy if the participant’s spouse were to talk to men outside the family.

**Table 2:** Multivariable analysis exploring correlates of DV perpetration among newly-married men living in Pune slum communities (n = 100).

		Correlation with Past 3-month perpetration of DV					
		Full combined model		Reduced combined model		Final model	
Domain	Predictor	Stand. β	p-value	Stand. Β	p-value	Stand. Β	p-value
Control vars.	Age					-0.044	Ns
	Education					-0.080	Ns
Socio-demographics	No. of Household members	0.124	0.165				
DV conceptualization and acceptance	Situational acceptance of wife’s sexual refusal	-0.092	0.288				
	Acknowledgment of items constituting DV	-0.231	0.017	-0.235	0.015	-0.217	0.027
Marital relationship and marital family relationship	Time spent with partner alone each week post-marriage	-0.185	0.033	-0.221	0.009	-0.230	0.007
	Prioritization of time spent working toward goals of self	0.081	0.355				
	Extent of attainment of the “husband ideal”	-0.189	0.028	-0.206	0.013	-0.201	0.017
	Extent of spouse’s attainment of the “wife ideal”	-0.058	0.521				
	Extent of jealousy if spouse talks to men outside family	0.221	0.016	0.274	0.002	0.272	0.002
Sexual communication and behaviors, and sexual and reproductive health	Capacity to communicate unwillingness to have sex with partner	-0.034	0.701				
Stress, resilience, and social support	Resilience	-0.228	0.029	-0.288	0.002	-0.304	0.002

Stand = standardized; vars = variables.

## Discussion

This study is the first to examine drivers of DV perpetration in slum communities in India and contributes to the global literature that informs strategies that engage men to prevent DV in resource-limited settings. Among recently-married men residing in Pune slums, past 3-month DV perpetration was associated with low resilience, perception of not having achieved the “husband ideal,” conservatively defining DV, jealousy, and less time spent alone in the relationship. While others have not directly examined whether DV perpetration is associated with low resilience, prior studies have shown an inverse relationship between social support and coping and DV perpetration.[45] Similarly, our finding jealousy to be associated with DV perpetration is consistent with the literature.[31] Although other studies have not examined whether DV is associated with self-perceived shortcomings in achieving the “gender ideal,” studies examining the link between perpetration and low self-esteem have found little to no evidence in support of the association.[31] Differences in our study findings versus the others (which were conducted in high-income settings) may be due the forces of patriarchy and subordination being stronger on our participants, who comprise one of the lowest socio-economic strata of India. While several studies have examined the effect of relationship discord on DV, we only found one study that examined the link between DV and time spent in the relationship.[46] This study, unlike ours, found no evidence of an association; however, it only assessed the link between time spent and sexual DV and explored DV experience by recently-married women in rural settings. To the best of our knowledge, no other studies have directly explored whether men’s conceptualization of DV directly impacts their perpetration of abuse. However, several studies have looked downstream and shown DV perpetration to be associated with attitudes condoning DV.[47,48] Personal experiences of violence (i.e. abuse as a child, witnessing an abusive relationship among one’s parents) likely shape how one defines DV and perceives DV-induced harm and attitudes toward its acceptance.

Many determinants of DV identified in the existing literature were not significant in this population. For example, unlike many studies in India [49], socio-economic factors (including gaps in age, education, and income—data not shown) were not associated with DV. This is likely due to the relative homogeneity of our study population with regard to these factors. Unlike prior research suggesting pregnancy to have a protective effect on DV[50], pregnancy was not associated with less perpetration in our study. Perhaps, this was because the duration of childlessness was insufficient to challenge masculinity or to result in pressure from others, as commonly happens if a child is not born within the initial years of marriage. Consistent with the evidence examining the link between arranged versus love marriages on DV, the level of premarital acquaintance was also not associated with DV. This finding is in part comforting, because it suggests post-marital interventions can reduce DV in the context of both love and more common, traditional arranged marriages (in which the couple meets minimally in the pre-marital period). It is important to note that less modifiable familial factors (i.e. satisfaction with dowry and daughter-in-law) fell out of the final model, suggesting that interventions that focus on the relationship could prevent DV without having to address these factors. Lastly, while resilience was protective, stress was not associated with DV perpetration. This may be because survey items (although comprehensive in the extent and types of stress examined) measured stress perceived versus stress experienced.

The key strengths of this study include that is one of a handful of studies globally that explores determinants of DV perpetration in an LMIC, and particularly, in one of the most DV vulnerable subpopulations. Further, it is unique in that it looks at risk factors for early marital DV perpetration, whereas most studies to date focus on lifetime DV or past-year DV at any time in the course of a marital relationship. Additionally, we explored an exhaustive list of potential socio-cultural and behavioral correlates that were derived from the literature and tailored to the Indian context and our outcome measure of DV perpetration was measured using a scale derived and validated in the same community. In doing so, we uncovered novel, modifiable risk factors for DV perpetration. Lastly, this study sheds critical light on a very difficult-to-reach population. For example, the participants’ time availability was limited due to long working days and social norms encouraging alcohol and socialization by night, their mothers often discouraged their participation, and private, convenient venues for conducting interviews were difficult to come by in the communities in which the participants lived.

A significant limitation of the study is its cross-sectional design which hinders our ability to draw causal inferences. While we hypothesize that conservative conceptions of behaviors constituting violence, perception of not achieving the “husband ideal,” low resilience, increased jealousy, and less time together in the relationship led to increased DV perpetration, the converse may also be true. It is possible that perpetration of abuse and not recognizing the due harm could have led men to have more conservative definitions of DV. Similarly, perpetrating DV could have led men to feel they were betraying the “husband ideal,” to have a lower self-esteem and weakened support system, which in turn negatively impacted their resilience. It is also possible that DV perpetration led to his spouse spending less time with him alone, poorer relationship quality, and thus increased jealousy. Another limitation of the study was the restriction of the sample to slums in Pune, which may limit generalizability of findings to slums in other parts of India and abroad. Also, this study was originally designed to inform the development of a DV intervention and not specifically powered to detect differences in DV. While the high frequencies of DV perpetration in this sample provided the opportunity for this unique analysis, future studies should validate these results on a larger scale. Lastly, while it would have been opportune to link the determinants of DV experience noted in the women’s survey to that of DV perpetration in the men’s survey, research ethics concerning her safety prohibited our surveying participants’ spouses.

In conclusion, our study is the first to exhaustively explore socio-behavioral correlates of early marriage DV perpetration in a low-income community in an LMIC. Future studies should explore in-depth the pathways and direction of association between the five correlates identified in the multivariable analysis and DV perpetration. Further, our findings should inspire primary DV prevention interventions that engage either the couple as a unit or men as potential perpetrators. Both such strategies should consider incorporating components to build resilience and self-esteem, challenge men to expand their definitions of DV, and enable them to prioritize spending time together alone with their spouse.

## Supporting information

S1 Appendix Survey instrument. (PDF)
